# Project control- evaluation of a brief HIV counseling video to improve risk reduction behavior of teenagers

**DOI:** 10.1186/1742-4690-9-S1-P61

**Published:** 2012-05-25

**Authors:** Yvette Calderon, Cheng-Shiun Leu, Ethan Cowan, Jillian Nickerson, Christopher Brusalis

**Affiliations:** 1 Albert Einstein College of Medicine, Bronx, NY, USA; 2 Columbia University, New York, NY, USA; 3 Jacobi Medical Center, Bronx, NY, USA

## Background

This study compared the effectiveness of a brief theory-based, youth-friendly HIV counseling video series with the standard practice (an HIV counselor) in improving risk reduction behavior among teens recruited in an urban Emergency Department (ED).

## Methods

A two-armed randomized controlled trial was conducted on a convenience sample of 203 non-critically ill, sexually active individuals aged 15-21 in an urban emergency department. Participants in the control (counselor) group received HIV information and counseling from a counselor while those in the intervention (video) group watched a series of youth-friendly counseling videos tailored to patients’ stages of change. All participants completed pre- and post-intervention measures on three mediating variables hypothesized to reduce unsafe sexual behavior: condom intention, condom outcome expectancy, and condom self-efficacy. HIV testing was optional for both arms.

## Results

203 patients were enrolled and randomized, 102 in the video group and 101 in the counselor group. The groups were similar with respect to age, gender, race, ethnicity, and sexual history. The video intervention performed as well as in-person counseling at improving several condom use measures. The mean difference between groups (video-counselor) in improvement over time (from pre- to post-counseling) in condom self-efficacy was 0.26, CI(0.03,0.50), in male outcome expectancy was 0.15, CI(0.02,0.28), and in female outcome expectancy was 0.20, CI(-0.01,0.40). Participants in the video group improved their condom use intention score significantly more than those in the counselor group, with a mean difference between arms for change over time of 1.02, p-value= 0.01, CI(.24,1.80). The intervention effect on condom intention score did not differ by gender or ethnicity.

## Conclusions

The use of theory-based, youth-friendly video can be a valid means to provide post-test counseling education and prevention messages within an urban ED. The theory-based prevention messages can improve specific mediators representing risk reduction behavior among teenagers immediately following the intervention.

